# Supervised exercise training reduces oxidative stress and cardiometabolic risk in adults with type 2 diabetes: a randomized controlled trial

**DOI:** 10.1038/srep09238

**Published:** 2015-03-18

**Authors:** Giovanni Vinetti, Chiara Mozzini, Paolo Desenzani, Enrico Boni, Laura Bulla, Isabella Lorenzetti, Claudia Romano, Andrea Pasini, Luciano Cominacini, Deodato Assanelli

**Affiliations:** 1 Department of Clinical and Experimental Sciences, University of Brescia, Brescia, Italy; 2 Department of Medicine, Section of Internal Medicine, University of Verona, Verona, Italy; 3 Diabetology Unit, Azienda Ospedaliera Spedali Civili di Brescia, Montichiari, Italy; 4 1^st^ Division of General Medicine, Azienda Ospedaliera Spedali Civili di Brescia, Brescia, Italy

## Abstract

To evaluate the effects of supervised exercise training (SET) on cardiometabolic risk, cardiorespiratory fitness and oxidative stress status in 2 diabetes mellitus (T2DM), twenty male subjects with T2DM were randomly assigned to an intervention group, which performed SET in a hospital-based setting, and to a control group. SET consisted of a 12-month supervised aerobic, resistance and flexibility training. A reference group of ten healthy male subjects was also recruited for baseline evaluation only. Participants underwent medical examination, biochemical analyses and cardiopulmonary exercise testing. Oxidative stress markers (1-palmitoyl-2-[5-oxovaleroyl]-sn-glycero-3-phosphorylcholine [POVPC]; 1-palmitoyl-2-glutaroyl-sn-glycero-3-phosphorylcholine [PGPC]) were measured in plasma and in peripheral blood mononuclear cells. All investigations were carried out at baseline and after 12 months. SET yielded a significant modification (p < 0.05) in the following parameters: V'O_2max_ (+14.4%), gas exchange threshold (+23.4%), waist circumference (−1.4%), total cholesterol (−14.6%), LDL cholesterol (−20.2%), fasting insulinemia (−48.5%), HOMA-IR (−52.5%), plasma POVPC (−27.9%) and PGPC (−31.6%). After 12 months, the control group presented a V'O_2max_ and a gas exchange threshold significantly lower than the intervention group. Plasma POVC and PGPC were significantly different from healthy subjects before the intervention, but not after. In conclusion, SET was effective in improving cardiorespiratory fitness, cardiometabolic risk and oxidative stress status in T2DM.

Physiological levels of reactive oxygen species (ROS) are important to maintain various cell functions, although an overload of ROS that exceeds the capacity of the antioxidant system can induce oxidative stress^1^. Oxidative stress plays a key role in both initiation and complications of type 2 diabetes mellitus (T2DM)^2^. The phospholipid 1-palmitoyl-2-arachidonyl-sn-glycero-3-phosphorylcholine (PAPC) is a major component of cell membranes and lipoproteins. Oxidation products of PAPC (lumped together under the abbreviation oxPAPC) are found in cells during inflammation, in membranes of apoptotic cells, as well as in oxidized low density lipoproteins and are considered sensitive markers of systemic oxidative stress^3^. oxPAPC can be isolated directly from plasma or from peripheral blood mononuclear cells (PBMC). Plasma oxPAPC comes from lipoproteins and fragments of apoptotic cells, while PBMC oxPAPC originates from incorporation into cell membranes and is used as in vivo surrogates of endothelial cells^3^. Furthermore, it has been demonstrated that ROS generation from mononuclear cells in response to hyperglycemia may contribute to a proinflammatory state that induces insulin resistance, even in the absence of increased abdominal adiposity^4^.

Cardiorespiratory fitness is the ability to transfer oxygen from ambient air to skeletal muscle mitochondria during sustained exercise with large muscle groups, whose criterion measure is the maximal oxygen consumption (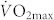), a concept that implies a precise interplay between pulmonary, cardiovascular and neuromuscular apparatuses^5^. A low cardiorespiratory fitness represents a greater risk factor than obesity for the development of type 2 diabetes mellitus (T2DM)^6^ and subjects with T2DM, in the absence of complications, have a decreased exercise performance compared to healthy subjects^7^. In addition, an exercise intervention *per se* can improve blood glucose control and cardiovascular risk in T2DM^8^,^9^, especially if a combination of aerobic and resistance training is performed regularly and for a long period of time^10^. To mechanistically explain these observations, it has been hypothesized that endurance training enhances antioxidant capacity^11^,^12^,^13^ and reduces systemic low-grade inflammation^14^. This is particularly evident in mononuclear cells of insulin-resistant obese subjects^15^ as well in subjects with T2DM^14^. As a consequence, beta-cell function, insulin sensitivity and vascular function are supposed to improve^14^.

Cardiopulmonary exercise testing (CPX) is the preferred tool to assess cardiorespiratory fitness and it is increasingly being used in a wide spectrum of clinical conditions affecting exercise capacity^16^. Although most studies on exercise in T2DM used CPX^9^,^17^,^18^, endurance exercise prescription was based on a fixed fraction of 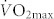 or of maximal heart rate. Given that these methods may have significant individual standard deviation, we believe that a direct estimation of heart rate at ventilatory thresholds would detect more accurately the optimal training intensity^19^.

Previous studies on the effects of exercise in T2DM were based mostly on short-term interventions, with mean duration of 15–24 weeks^9^,^17^,^18^,^20^,^21^,^22^. In this study, we tested the hypothesis that a 12-months supervised exercise training intervention on subjects with T2DM can positively affect three major indicators: oxidative stress markers, cardiorespiratory fitness and cardiometabolic risk.

## Methods

We conducted a clinical trial involving the Sport and Exercise Medicine Centre and the Diabetology Service, “Spedali Civili di Brescia” Hospital Trust, Hospital of Montichiari, Italy.

### Participants

To avoid confounding factors that could affect oxidative stress status and cardiometabolic risk, we selected only male subjects, aged between 40 and 70 years, nonsmokers and not taking antioxidant supplements. Twenty subjects with T2DM were recruited and randomized into an intervention group that performed supervised exercise training for one year (SET group; n = 10) and a control group (C group; n = 10) that received standard medical care only. Specific eligibility criteria included BMI between 25 and 34.9 (overweight and grade I obesity), diagnosis of T2DM^23^ for at least 2 years, metabolic syndrome phenotype^24^, no need for insulin therapy, arterial hypertension and dyslipidemia controlled by statins and either ACE-inhibitors or angiotensin receptor blockers, absence of diabetes-specific complications and ischemic heart disease. A third group of ten healthy individuals (H group; n = 10) was also recruited as a reference population and it was examined only at baseline without receiving any intervention. Eligibility criteria included BMI between 18.5 and 24.9 (normal weight), absence of any active medical condition and chronic medication prescription.

Research protocol was approved by the Ethics Committee of Spedali Civili di Brescia (12 May 2010) and participants gave written informed consent. All experiments were performed in accordance with the Declaration of Helsinki.

### Measurements

Investigations consisted of medical examination, biochemical analyses, systemic oxidative stress evaluation and CPX. C and SET groups were examined at baseline (T0) and after twelve months (T12), while H group only at T0.

Medical examination was intended to rule out any contraindication to a maximal exercise test and to assess blood pressure, waist circumference, BMI and dietary habits. Dietary intake was studied in SET and C group with the European Prospective Investigation into Cancer and Nutrition food frequency questionnaire^25^. The NAF software (Nutritional Analysis of Food Frequency Questionnaires, National Cancer Institute, Milan, Italy)^26^ was used to transform information about food composition into total energy intake.

Biochemical analyses were performed at the laboratories of “Spedali Civili di Brescia” Hospital Trust at T0 and T12. Venous blood samples were obtained from each subject at 8:00 a.m after at least 12 hours fasting and 48 hours from the last exercise bout; morning doses of antidiabetic medications were discontinued. The following parameters were evaluated: fasting plasma glucose, triglycerides, total and HDL cholesterol (Dimension Vista, Siemens Healthcare, Erlangen, Germany), HbA1c (Variant II Biorad Bio-Rad Laboratories, Hercules, California, USA) and serum insulin (ADVIA centaur IRI, Bayer Diagnostics Europe, Dublin, Ireland). Insulin resistance was calculated using the Homeostasis Model of Assessment - Insulin Resistance (HOMA-IR) formula [(serum insulin in mUI/l) × (fasting plasma glucose in mMol/l)/22.5]. LDL cholesterol was estimated by the Friedewald formula. In addition, metabolic syndrome severity score (Z-score) was calculated starting from individual components of metabolic syndrome^27^.

The research laboratories of the University Hospital of Verona, Italy, evaluated systemic oxidative stress by measuring oxidized forms of the phospholipid 1-palmitoyl-2-arachidonyl-sn-glycero-3-phosphorylcholine (ox-PAPC). The following different oxPAPC were taken into consideration: 1-palmitoyl-2-(5-oxovaleroyl)-sn-glycero-3-phosphorylcholine (POVPC); 1-palmitoyl-2-glutaroyl-sn-glycero-3-phosphorylcholine (PGPC). Plasma and peripheral blood mononuclear cells (PBMC) concentrations of oxPAPC at T0 and at T12 were tested. After appropriate storing and carriage, samples were drawn into pyrogen-free blood collection tubes. Multiple aliquots of serum were placed into sterile 1-ml screw-capped polypropylene vials with phenolic antioxidant 2,6- Di-tert-butyl-4-methylphenol 10 mmol/L (Sigma) added to inhibit lipid peroxidation and stored at 280 uC. Samples were frozen and thawed only once. PBMC were isolated as previously described^28^. Briefly, whole blood was layered onto a sterile aqueous medium containing Ficoll and sodium diatrizoate at a predetermined density of 1.007 g/ml at 25 uC. Gentle centrifugation at room temperature resulted in separation of PBMC at the blood/ficoll interface, with other white and red blood cells passing through the interface. OxPAPC in PBMC and plasma of patients were measured on an Agilent mass spectrometer equipped with an electrospray source as previously described^29^. Flow injection experiments were performed by an HPLC system (HP1100; Agilent Technologies). Quantification of peak areas was performed by single ion monitoring in the elution time range of 10–20 min using appropriate software. Authentic1-palmitoyl-2-arachidonoyl-sn-glycero-3-phosphocholine (PAPC), POVPC and PGPC were obtained from Avanti Polar Lipids, Inc. (Alabaster, AL).

CPX was performed on an electro-magnetically braked cycle ergometer (Cardioline, Italy), with an incremental ramp protocol (20 W/min) until volitional exhaustion or an evident plateau in the 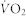 versus work rate curve. Expired gases and volumes were measured by an automated breath-by-breath respiratory gas analysis system (Ultima CPX, Medical Graphics, USA). The Breeze Suite software (Medical Graphics, USA) allowed to interpolate breath-by-breath gas data to 1-s values with the preventive exclusion of outlying breaths (> ±3 SD from the adjacent five breaths) and to determine gas exchange thresholds with computerized linear regression analysis (V-slope method)^30^. Gas exchange threshold (GET) was defined as the point where the slope of the 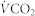 versus 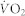 curve increases^31^, while the ventilatory compensation point (VCP) as the increase of the slope of the 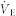 versus 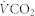 curve^32^. Digital 12-lead ECG was simultaneously recorded (Cardioline, Italy), sending to the previous device information about heart rate. Blood pressure and pulse oximetry were also monitored during the test. Outcome measures were 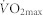, 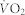 and heart rate at GET (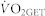, HR_GET_) and at VCP (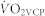, HR_VCP_).

### Interventions

All subjects with T2DM received standard medical care aimed at achieving optimal glycemic, lipid, blood pressure and body weight targets, as established by existing guidelines^23^, including glucose-, lipid- and blood pressure-lowering agents and a dietary regimen prescribed by the diabetologist. No further nutritional intervention was given throughout the study. Medications were adjusted throughout the study to account for potential reduced needs. Subjects in C group did not receive any type of intervention other than standard medical care. H group did not receive neither intervention nor follow-up.

SET group's training program consisted of 12 months of aerobic, resistance and flexibility training, according to the most recent guidelines^10^. Training sessions were performed in a hospital-based setting and supervised by personal trainers with specialist degree in “preventive and adaptive physical activity”. Global weekly workload was gradually increased from 140 to 270 minutes. Endurance training involved cycling on mechanically braked cycle ergometers while wearing heart rate monitors, at the intensity individually prescribed according to the results of CPX. In the first two months, endurance training was performed approximately 5 bpm below HR_GET_. From the third month, training heart rates have been allowed to temporary increase above HR_GET_, gradually reaching but not overcoming HR_VCP_, in an interval-training fashion. Time per session has been increased progressively in the first 3 months, starting from 15 minutes and reaching the target of 35 minutes.

Resistance training consisted of 40 to 50 minutes of different exercises involving the major muscle groups (upper limb, lower limb, chest, back and core). Exercises consisted both in calisthenics and repetitions with ankle weights, dumbbells and elastic bands. Subjects began with 3 sets of 8 repetitions, then progressively improved to 3 sets of 12–15 repetitions. For exercises requiring dumbbells, weights started from 1–3 kg and were increased up to 2–6 kg depending on the subject and the type of exercise. Flexibility training was composed of static stretching exercises that involved upper and lower body, before and after the resistance training sessions.

### Statistical Analysis

Baseline to end-of-study changes (expressed as mean ± standard deviation) were analyzed using Student's t-test for paired samples. To assess the statistical significance of differences between groups at each time point we used Student's t-test for independent samples. A value of *p* < 0.05 was considered statistically significant.

## Results

### Baseline (T0)

Mean age ± standard deviation of SET, C, and H group was respectively 60.56 ± 5.94, 57.50 ± 9.46 and 54.6 ± 8.7 years, with no significant difference. Among subjects with T2DM, 13 (7 in SET and 6 in C) took metformin 1.5–2.5 g/day, 4 (1 in SET and 3 in C) took metformin 1.7 g/day plus pioglitazone 30 mg/day, 3 (2 in SET and 1 in C) took metformin 2 g/day plus glimepiride 3 mg/day. As a consequence of inclusion criteria, all patients in SET and C group took a statin and either an ACE-inhibitor or an angiotensin receptor blocker.

**Figure 1:**
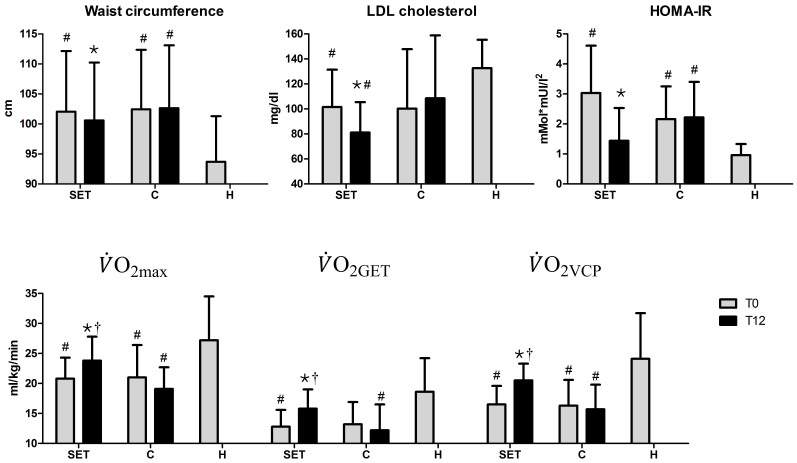
The most significant cardiometabolic and cardiorespiratory parameters at baseline (T0) and at the end of the study (T12). SET: supervised exercise training. C: control. H: healthy. 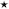: *p* < 0.05 vs. T0; †: *p* < 0.05 vs C at T12; #: *p* < 0.05 vs H.

Cardiometabolic risk, cardiorespiratory parameters and total energy intake did not differ significantly between SET and C groups, while in H group felt within the range of normality (Figure 1 and Table 1). Mean 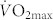, adjusted for body weight, was nearly 21 ml·kg^−1^·min^−1^ for both groups. H group had a higher maximal aerobic power in comparison SET and C group (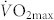 27.2 ml·kg^−1^·min^−1^, *p* = 0.03 and *p* = 0.04, respectively) and a higher 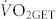 and 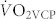 (*p* = 0.006 and *p* = 0.003 respectively). In all participants HR_max_ and maximal work rate corresponded to more than 85% of predicted value based on an age- and sex-matched population, concomitant exercise ECG was negative for ischemia and there was no arterial oxygen desaturation during effort. In SET group, mean HR_GET_ was 116 bpm, while mean HR_VCP_ was 129 bpm. Mean endurance exercise intensity prescription was 79% of the measured HR_max_ (111 bpm, that is HR_GET_ – 5 bpm) for the first two months and between 79% and 92% of HR_max_ since the third month, when interval training was started. These values correspond to 69%–87% of the HR_max_ estimated with the formula 220 – age.

**Figure 2:**
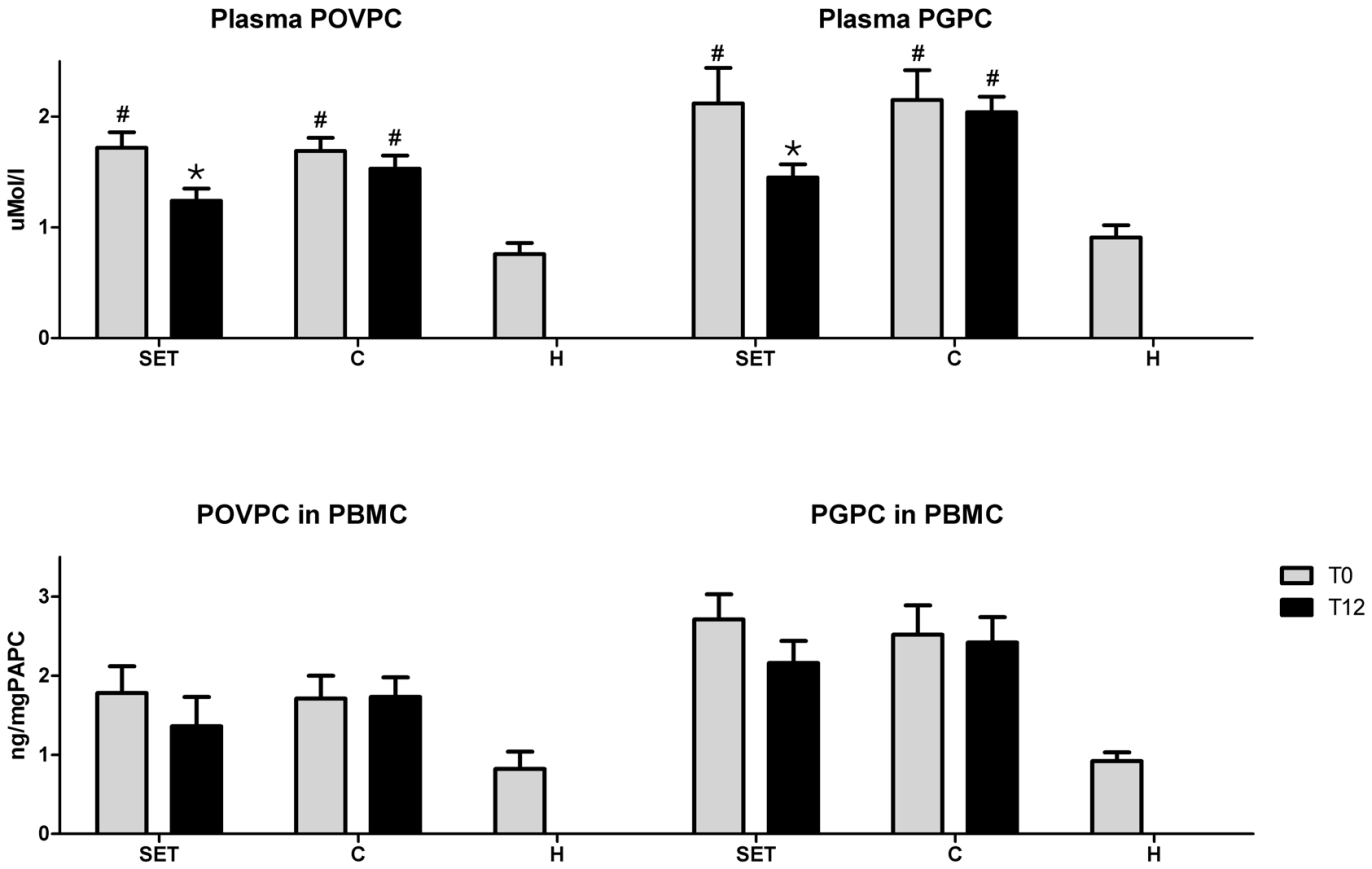
1-palmitoyl-2-(5-oxovaleroyl)-sn-glycero-3-phosphorylcholine (POVPC) and 1-palmitoyl-2-glutaroyl-sn-glycero-3-phosphorylcholine (PGPC) levels in plasma and in peripheral blood mononuclear cells (PBMC) at baseline (T0) and at the end of the study (T12). SET: supervised exercise training. C: control. H: healthy. 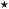: *p* < 0.05 vs. T0; #: *p* < 0.05 vs H.

oxPAPC levels are shown in Figure 2. At baseline SET/C group presented plasma concentrations of POVPC and PGPC respectively 226%/212% and 232%/224% higher respect to H group (*p* = 0.02/*p* = 0.01 and *p* = 0.01/*p* = 0.02 respectively). No statistically significant variation are detected in PBMC (Figure 2), but the trend is similarly towards higher levels among SET and C groups compared to H (*p* = 0.21/p = 0.34 and *p* = 0.51/*p* = 0.14).

### End of the study (T12)

There was no drop-out in either group. In SET group, the two patients on metformin 2 g/day plus glimepiride 3 mg/day had the dose reduced by diabetologist to metformin 2 g/day plus glimepiride 1 mg/day because of recurrent hypoglycemia at self-monitored capillary glucose. 24-hour recall at T12 showed no significant variation of total energy intake (Table 1). SET group obtained a significant improvement in several cardiometabolic parameters (Figure 1). Waist circumference changed from 102.1 ± 10.1 cm to 100.6 ± 9.6 cm (−1.4%, *p* = 0.01), LDL cholesterol from 102 ± 30 to 81 ± 24 mg/dl (−20.2%, *p* = 0.04), HOMA-IR from 8.22 ± 5.59 to 4.22 ± 3.19 (−52.5%, *p* = 0.02) and similarly fasting insulinemia from 8.22 ± 5.59 to 4.22 ± 3.19 uUI/ml (−48.5%, *p* = 0.01). There was a trend towards lower body weight, BMI, HbA1c and metabolic syndrome severity score (Table 1). Conversely, in C group there was no significant change in any metabolic parameter during the observation period. Compared to C group, SET group obtained a non-significantly lower total and LDL cholesterol, fasting insulinemia, HOMA-IR, triglycerides, systolic blood pressure, diastolic blood pressure and metabolic syndrome severity score (Z-score).

For what concerns cardiorespiratory parameters, SET group showed significant improvements (Figure 1). Absolute and weight-adjusted 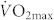 increased by 10.6% and 14.4%, respectively (*p* = 0.04 and *p* = 0.03), reaching a weight-adjusted value of 23.8 ml·kg^−1^·min^−1^. Higher values of absolute and weight-adjusted 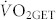 (+25.0% and + 23.4%, *p* = 0.02 and *p* = 0.01 respectively) and 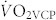 (+20.3% and + 24.2%, *p* = 0.005 and *p* = 0.007, respectively) were also found, without a significant change in the corresponding HR_GET_ and HR_RC_. Work rate at GET, work rate at VCP and maximal work rate significantly improved, while HR_max_ did not vary significantly (Table 1). C group did not show any significant change in cardiorespiratory parameters during the observation period, although there was a non-significant decrease in 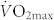 from 21.0 ± 5.4 ml·kg^−1^·min^−1^ to 19.1 ± 3.6 ml·kg^−1^·min^−1^ (−9.0%). SET group achieved an absolute and weight-adjusted 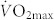 16.6% (*p* = 0.04) and 24.6% (*p* = 0.01) greater, respectively, compared to C group. SET group achieved also a higher weight-adjusted 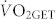 and 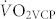 (+29.5% and + 30.6%, respectively, *p* = 0.03 for both) and achieved a significantly higher maximal work rate (Table 1 and Figure 1). At T12, weight-adjusted 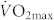 of SET group was no more significantly different from that of H group, while that of C group still remained lower (*p* = 0.0042).

For what concerns oxPAPC (Figure 2), SET group obtained a significant decrease of plasma concentrations of POVPC and PGPC at T12 (−27.9%, *p* = 0.03 and−31.6%, *p* = 0.04 respectively) respect to C who obtained a decrease of 10% or lower (*p* = 0.76 and *p* = 0.65, respectively). A statistically significant decrease was not achieved in PBMC, but the trend was similar to that of plasma.

## Discussion

Our data confirms that subjects with T2DM present a more oxidizing environment than healthy subjects^2^ and the significant decrease of plasma oxPAPC concentrations in SET group indicates that this specific exercise program has had a role in the amelioration of oxidative stress status. It must be taken into account that the following considerations are limited to male sex, since women were not included. It is hereby argued that reduction of plasmatic phospholipids can be due to two major mechanisms: increased antioxidant defense and reduced ROS production. Increased antioxidant defense may involve an exercise-induced stimulation of the antioxidant response pathway. For this purpose it would be of interest to study the transcription factor NF-E2-related factor 2 (Nrf2), which is responsible for the expression of antioxidant response genes. It has been demonstrated that in T2DM there is a deregulated Nrf2-dependent antioxidant defense pathway with increased inflammatory status and it has been shown that antioxidant activity is enhanced by exercise and increases in response to endurance training^12^,^13^. Decreased ROS production may involve a reduced substrate overload in mitochondria, either thanks to a decreased blood glucose and LDL concentrations or a training-induced improved mitochondrial function. A limitation of this study is the paucity of different ROS-modified biomolecules investigated. Lipid peroxidation could have been more deeply evaluated with detection of additional lipid hydroperoxides^3^, whereas oxidative modification of proteins could have been investigated with dosage of protein carbonyls and nytrotirosin^33^. Actually, all these markers could be sensitive to a lifestyle intervention^33^,^34^. The absence of statistically significant changes in PBMC could be due to the low numerousness of the sample, to a possible low intensity of the training program (as discussed below) and to the lack of a clinically meaningful weight loss. Furthermore, the absence of diabetes-specific complications and the good blood glucose control (mean HbA1c values were <7.0%) are favorable factors that could protect circulating cells without allowing accumulation of oxidative products.

Since evidence for oxidative stress as a key mechanism altering insulin resistance has been widely discussed^2^,^4^, the significant reduction HOMA-IR in SET group confirms this pathogenetic event and suggests that it can be partially reversed. From a molecular point of view, there is evidence that ROS contribute to insulin resistance and to the activation of pro-inflammatory signaling pathways, mainly regulated by the transcription factor kB (NF-kB)^35^. Conversely, insulin has a strong anti-inflammatory effect, including a reduction in intranuclear NF-kB and decrease of ROS generation^36^, so it is also possible that the improvement of HOMA-IR was at least partially sustained by the reduction of systemic oxidative stress. Unfortunately, HOMA-IR is not the gold standard for the quantification of insulin resistance, so caution is needed in interpreting these results. Another limitation is not having taken into account post-prandial changes in oxidative stress markers, which would have provided more understanding of exercise-diet interactions. Since glucose fluctuations exhibited a more specific triggering effect on oxidative stress than chronic sustained hyperglycemia^37^, it could be stimulating to evaluate also intraday glycemic variability with continuous glucose monitoring systems.

Previous studies and meta-analyses showed that structured exercise training improves physical fitness in subjects with T2DM, along with a reduction of HbA1c^9^,^21^. In our study we found a non-significant reduction of HbA1c (*p* = 0.08), despite a significant improvement of HOMA-IR. This is probably due to the low number of subjects enrolled in SET group (n = 10), the mean baseline HbA1c value <7.0% (which suggests optimal diabetes control), the decrease of ant-diabetic medications in two subjects and the absence of a specific nutritional intervention.

Despite a slight decrease of body weight was observed in all groups, this difference is not statistically significant, while a significant reduction in waist circumference has been observed (−1.5 cm). This is consistent with other studies, where an increase in fat free mass and a decrease in fat mass is reported without changes in total body weight^38^. Supervised exercise training produced a significant reduction of LDL cholesterol in subjects already on statins, not observed in previous trials on T2DM^8^. However, the significant reduction in body fat^39^ or even the training alone^40^ could be responsible for this finding. Variations in adherence to statin therapy is also to be taken in consideration.

Our findings strengthen the theory that subjects with T2DM and overweight have decreased exercise capacity compared to healthy subjects^7^. In fact, at baseline both SET and C group presented a low 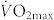. The hypothesis to explain this finding are various in literature: advanced age, increased BMI, poor diabetes control, left ventricle diastolic dysfunction and diabetic neuropathy^7^. In our subjects, we hypothesize that the low exercise capacity is mainly due to a very important cardiovascular and muscular deconditioning and to an increased body mass, both likely caused by several years of sedentary lifestyle. In fact, 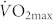 substantially improved after one year of reconditioning (23.8 ml·kg^−1^·min^−1^). We want to emphasize that 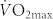, in addition to his physiological importance, represents a prognostic index that is inversely related to cardiovascular and all-cause mortality^41^ and should not be overlooked when estimating cardiometabolic risk.

It is to be remarked that all subjects with T2DM were on metformin, a drug that has been found to blunt several effects of exercise, like improved cardiorespiratory fitness^42^ and in insulin sensitivity^43^, although there are conflicting findings^44^. In our study, we cannot exclude these detrimental effects, given the limited cardiometabolic responses despite the long duration of the training program. It has also been demonstrated that, by increasing heart rate, metformin could lead to the prescription of a lower endurance exercise intensity^45^. We believe that this is not occurred in our study, since the direct assessment HR_GET_ and HR_VCP_ with CPX permits to bypass this effect, in analogy to what happens with beta blockers. A theoretical risk of sub-optimal endurance intensity prescription is in any case present in the later phases of the intervention, since our study lacks of an intermediate CPX to better adapt the progression of training to individual physiological adaptations.

Along with cardiorespiratory fitness, increased strength plays an important role in intramuscular glucose and fat uptake and oxidation, contributing along with cardiorespiratory fitness to an increased insulin sensitivity and a decreased visceral fat in subjects with T2DM^10^. In our study, resistance exercise prescription was less standardized than endurance, with a drift towards lighter weights, maybe explaining some blunted cardiometabolic and antioxidant adaptations to training.

The long-term compliance of SET group subjects deserves a special mention, as no one dropped out from the study. This might be possible thanks to a personalized approach, a protected training environment, the supervision of qualified exercise professionals and the gratuitousness of the service offered. In contrast, physical activity advice alone or theory-based intervention gathered only poor results^22^,^46^.

In conclusion, this study demonstrates that personalized and supervised exercise training of 1-year duration can affect positively insulin sensitivity and blood levels of LDL cholesterol. Increased cardiorespiratory fitness and a healthier body composition are probably the underlining causes. All these factors, with complex interactions, might have brought to a reduced systemic oxidative stress, as revealed by plasmatic oxPAPC. Moreover, the reduction of systemic oxidative stress may have had a positive feedback on insulin sensitivity. This provides new insights into the molecular mechanisms of non-pharmacologic treatment of chronic diseases.

## Author Contributions

G.V. and C.M. wrote the manuscript. G.V., D.A. and E.B. designed and performed the clinical test on subjects. P.D. and D.A. selected and examined the subjects. L.B. and I.L. designed and supervised the exercise training program. C.M., A.P., C.R. and L.C. designed and performed the oxidative stress markers dosage. All authors reviewed the manuscript.

## Figures and Tables

**Table 1:** Cardiometabolic and cardiorespiratory parameters (mean ± SD) at baseline (T0), at the end of the study (T12), differences within groups (Δ_T12-T0_) and between groups [SET vs C (*p*)]

	SET				C				SET vs C *(p)*			H
	T0	T12	Δ_T12-T0_ (%)	*p_T12 vs T0_*	T0	T12	Δ_T12-T0_ (%)	*p_T12 vs T0_*	T0	T12	Δ_T12-T0_	T0
Age (years)	60.56 ± 5.94	61.56 ± 5.94	1 ± 0 (1.6 ± 0%)	*-*	57.50 ± 9.46	58.50 ± 9.46	1 ± 0 (1.7% ± 0)	-	0.43	0.43	-	54.60 ± 8.73
Body weight (kg)	83.68 ± 10.53	80.97 ± 11.28	−2.71 ± 1.41 (−3.2 ± 1.7%)	0.08	85.06 ± 9.25	84.37 ± 7.95	−0.69 ± 2.05 (0.8 ± 2.4%)	0.32	0.71	0.65	0.06	75.80 ± 11.13
BMI (kg/m^2^)	29.65 ± 4.08	28.69 ± 4.35	−0.96 ± 1.47 (−3.2 ± 4.9%)	0.07	29.20 ± 3.11	28.95 ± 2.70	−0.25 ± 0.73 (−0.8 ± 2.5%)	0.37	0.80	0.88	0.23	24.06 ± 1.62
Systolic blood pressure (mmHg)	129.5 ± 14.0	121.5 ± 17.2	−8.0 ± 24.4 (−6.2 ± 18.8%)	0.33	133.6 ± 15.4	132. 5 ± 6.5	−1.1 ± 13.3 (−0.8 ± 9.9%)	0.82	0.56	0.11	0.48	114.0 ± 7.7
Diastolic blood Pressure (mmHg)	82.0 ± 9.5	77.5 ± 8.2	−4.5 ± 11.2 (−5.5 ± 13.6%)	0.23	84.0 ± 5.7	83.7 ± 4.6	−0.3 ± 7.1 (0.4 ± 8.4%)	0.92	0.61	0.07	0.36	78.0 ± 6.7
Total cholesterol (mg/dl)	172.78 ± 30.14	147.56 ± 31.85	−25.22 ± 29.9 (−14.6 ± 17.3%)	*0.03*	171.25 ± 47.63	178.62 ± 51.30	7.37 ± 30.7 (4.3 ± 17.9%)	0.52	0.94	0.15	0.05	211.9 ± 26.3
HDL cholesterol (mg/dl)	46.78 ± 9.59	46.33 ± 13.11	−0.44 ± 4.85 (−0.9 ± 10.4%)	0.79	48.12 ± 5.49	44.37 ± 9.27	−3.75 ± 7.48 (−7.8 ± 15.5%)	0.20	0.73	0.73	0.29	57.40 ± 13.65
Triglycerides (mg/dl)	120.78 ± 39.82	95.89 ± 36.96	−24.89 ± 41.72 (−20.6% ± 34.5%)	0.11	113.50 ± 30.61	129.25 ± 39.27	15.75 ± 40.06 (13.9 ± 35.3%)	0.33	0.68	0.09	0.06	109.30 ± 44.34
Fasting plasma glucose (mg/dl)	150.44 ± 30.75	137.78 ± 16.35	−12.67 ± 35.83 (−8.4 ± 23.8%)	0.32	137.00 ± 24.63	128.87 ± 28.04	−8.12 ± 18.68 (−5.9 ± 13.6%)	0.26	0.34	0.42	0.75	94.20 ± 4.80
HbA1c (%)	6.77 ± 0.59	6.44 ± 0.33	−0.33 ± 0.49 (−4.9 ± 7.2%)	0.08	6.29 ± 1.00	6.65 ± 0.91	0.36 ± 0.82 (5.7 ± 13.0%)	0.25	0.24	0.53	0.05	5.43 ± 0.25
Fasting insulinemia (uUI/ml)	8.22 ± 5.59	4.22 ± 3.19	−4.00 ± 3.87 (−48.7 ± 47.1%)	*0.01*	6.62 ± 3.58	7.37 ± 4.53	0.75 ± 2.49 (11.3 ± 37.6%)	0.42	0.50	0.11	*0.02*	4.10 ± 1.37
Metabolic syndrome severity score (Z-score)	0.77 ± 0. 77	0.35 ± 0.67	−0.42 ± 0.59 (−54.5 ± 76.1%)	0.07	0.57 ± 0.56	0.68 ± 0.62	0.11 ± 0.44 (16.2 ± 77.2%)	0.52	0.57	0.31	0.06	−0.31 ± 0.12
Total energy intake (kcal/day)	2132 ± 26	2269 ± 62	157 ± 189 (6.4 ± 8.9%)	0.42	2150 ± 62	2193 ± 94	43 ± 101 (2.0 ± 47.0%)	0.46	0.87	0.25	0.43	2180 ± 72
Work rate at GET (watt)	107 ± 21	138 ± 17	31 ± 28 (29.0 ± 26.2%)	*0.01*	110 ± 25	111 ± 46	1 ± 33 (0.9 ± 30%)	0.94	0.79	0.12	0.05	145 ± 25
Work rate at VCP (watt)	134 ± 17	166 ± 19	32 ± 28 (23.9 ± 20.9%)	*0.008*	136 ± 33	138 ± 45	2 ± 33 (1.4 ± 24.3%)	0.85	0.91	0.10	0.05	178 ± 21
Maximal work rate (watt)	158 ± 16	179 ± 24	21 ± 24(13.3 ± 15.2%)	*0.03*	152 ± 36	154 ± 23	2 ± 26 (1.3 ± 17.1%)	0.85	0.66	*0.04*	0.13	190 ± 22
HR_GET_ (beat/min)	116 ± 12	116 ± 10	0 ± 15 (0 ± 12.9%)	0.98	116 ± 16	111 ± 15	−5 ± 9 (−4.3 ± 7.8%)	0.11	0.94	0.34	0.37	121 ± 15
HR_VCP_ (beat/min)	129 ± 13	132 ± 9	3 ± 14 (2.3 ± 10.9%)	0.54	129 ± 17	128 ± 16	−1 ± 9 (−0.8 ± 7.0%)	0.82	0.93	0.49	0.69	141 ± 11
HR_max_ (beats/min)	140 ± 12	141 ± 8	1 ± 8 (0.7 ± 5.7%)	0.75	139 ± 19	139 ± 20	0 ± 6 (0 ± 4.3%)	0.06	0.94	0.36	0.33	153 ± 13
